# Evidence and strategies for malaria prevention and control: a historical analysis

**DOI:** 10.1186/s12936-018-2244-2

**Published:** 2018-02-27

**Authors:** Gabriel Gachelin, Paul Garner, Eliana Ferroni, Jan Peter Verhave, Annick Opinel

**Affiliations:** 1 Laboratoire SPHere, UMR 7219, Université Paris Diderot. Sorbonne Paris Cité, 4, Rue Elsa Morante, 75013 Paris, France; 2 Centre for Evidence Synthesis in Global Health, Department of Clinical Sciences, Liverpool School of Tropical Medicine, Pembroke Place, Liverpool, L3 5QA UK; 3 Epidemiological Department of the Veneto Region, Passaggio Gaudenzio 1, 35131 Padua, Italy; 4 Dept. Med. Microbiology, Radboud University Medical Centre, Nijmegen, The Netherlands; 5 UMR 1181 Biostatistics, Biomathematics, Pharmacoepidemiology and Infectious Diseases, Institut Pasteur/Inserm/UVSQ, 25, Rue du Dr Roux, 75724 Paris Cedex 15, France

**Keywords:** History of malaria, History of vector control, House-proofing, Insecticide, Gambusia, Species sanitation, Marshes sanitation

## Abstract

Public health strategies for malaria in endemic countries aim to prevent transmission of the disease and control the vector. This historical analysis considers the strategies for vector control developed during the first four decades of the twentieth century. In 1925, policies and technological advances were debated internationally for the first time after the outbreak of malaria in Europe which followed World War I. This dialogue had implications for policies in Europe, Russia and the Middle East, and influenced the broader international control agenda. The analysis draws on the advances made before 1930, and includes the effects of mosquito-proofing of houses; the use of larvicides (Paris Green) and larvivorous fish (Gambusia); the role of large-scale engineering works; and the emergence of biological approaches to malaria. The importance of strong government and civil servant support was outlined. Despite best efforts of public health authorities, it became clear that it was notoriously difficult to interrupt transmission in areas of moderately high transmission. The importance of combining a variety of measures to achieve control became clear and proved successful in Palestine between 1923 and 1925, and improved education, economic circumstances and sustained political commitment emerge as key factors in the longer term control of malaria. The analysis shows that the principles for many of the present public health strategies for malaria have nearly all been defined before 1930, apart from large scale usage of pesticides, which came later at the end of the Second World War. No single intervention provided an effective single answer to preventing transmission, but certainly approaches taken that are locally relevant and applied in combination, are relevant to today’s efforts at elimination.

## Background

Laveran in Algeria discovered the cause of malaria and the *Plasmodium* parasite around 1880–1882. This was an important discovery, but did not at the time influence the various treatments and prevention approaches that were already in use. Nevertheless, understanding the biology of the diseases was clearly important to help develop strategies to intervene, and some 15 years later (around 1895–1899), Ross and Grassi defined the parasite’s life cycle and transmission by *Anopheline* mosquitoes. This understanding paved the way for a science-based strategy to tackle malaria by interrupting parasite transmission, or by tackling the insect vector, using tools already available. Control of malaria, even its eradication, seemed a possibility at the turn of the twentieth century.

At the time, it was known that quinine could kill the *Plasmodium* parasite in humans and it was a useful treatment. However, despite some promising initial results, using quinine to prevent transmission in large populations of people exposed to the risk of malaria was not successful at scale [1, 2].

Knowing that preventing people from being exposed to the vector opened the door to new strategies, such as reducing the vector through killing the larvae. From 1900 to 1925, success of local initiatives for malaria control were reported from a variety of areas where malaria was common, including Brazil, India, Italy, Palestine, Panama Canal and Spain, some of which are discussed later. Despite these apparent local successes, policy makers acknowledged the difficulty of interrupting malaria transmission, even in moderately high transmission areas. In the first comparative analyses of results in 1925 carried out by the League of Nations (the Malaria Commission of the League of Nations) and the First International Congress on Malaria, the authors concluded that none of the methods so far tested had proved efficient and stable enough to be confident that the answer to controlling malaria had been found [3, 4].

Indeed, these conclusions were probably reasonable. In contrast to the clinical use of quinine, it was not easy to make confident causal inferences about the effects of most of these interventions to control and prevent malaria at the population level. Multiple interventions were often poorly characterized and delivered without any formal comparison groups. Even the apparent effects from carefully documented, integrated interventions such as in the Canal Zone of Panama were not as clear as initially thought. The Zone was placed under a strict control as for yellow fever, leading to a marked reduction in the number of malaria cases (from 821/1000 in 1906 to 76/1000 in 1913 [5]); however, it appeared that this simply shifted the disease out of the area just adjacent to the Canal Zone to other places and populations of Panama, so that the final balance was not as positive as claimed. Indeed, medical entomologists showed that the diffusion of malaria in the construction area was due to human activities and attitudes [6]. On the whole, uncertainties and disputes around the effectiveness of the integrated interventions resulted from the lack of formal comparative studies, with inevitable confusion about which anti-malarial policies to apply, particularly at a large-scale level.

The year 1925 was auspicious as the first two international attempts to compare and discuss anti-malarial strategies took place. Firstly, the conclusions of the studies of anti-malaria policies used in Europe (including Russia) and the Middle East, which had been launched after the burst of malaria cases which followed WWI, were brought together and discussed at a special session of the malaria commission of the League of Nations, held in Geneva in 1925 [3]. Secondly, the First International Congress on Malaria, held in Roma in 1925 [7], discussed new anti-malarial strategies, particularly the use of larvicides such as Paris Green.

These two reports have been used in the present paper as a base for this analysis as they were important landmarks in the history of vector control. Over the 1899–1930 period of time trials or field operations were selected in which the authors report on a particular strategy or approach so that the evidence inferred could reasonably be attributed to the approach used. Studies were included that referred to in Europe and surrounding countries, particularly because a large body of well-defined data has been acquired at the very same time in geographical areas, mainly Southern Europe and the Middle East, which share numerous climatic and ecological features.

The analysis indicates that the logics underlying nearly all of the strategies presently used against malaria are those defined and tested during the first decades of the twentieth century. Also, the importance of combining different control measures to achieve successful control of malaria, was evident to 1925s malariologists, as appeared illusory the search for a unique way of control.

## Protecting houses and early domestic insecticides

At the very end of the nineteenth century, the world began to understand that the bite by a mosquito was more than just disagreeable-it was potentially dangerous. Thus, simple approaches to avoid mosquito bites took on a new meaning with the discovery of *Anopheles* as the vector of malaria, and were easily converted into methods to protect people from malaria, and potentially control transmission of the disease. Protection against bites with gloves and veiled hats, much in the same way as for bee-keepers, was widespread, and was recommended at night in some areas. Muslin mosquito nets, in use since the beginning of the eighteenth century, were sold as part of the routine equipment for outdoor recreational activities. Similarly, insect repellents, insecticides (pyrethrum) and sprayers, mosquito nets for cradles, were already available. Wire gauze was used for food-safe and windows. All those items could easily be found and even sold by mail-order catalogs, under the generic name of anti-flies and anti-mosquitoes or equipment for the “explorer” (see as an example, the catalog of the “*Manufacture des armes et cycles de Saint*-*Etienne*” 1895). Thus, the public had access to the basic materials to help reduce mosquito bites.

“Domiciliary malaria” [8] showed that most infections occurred from biting inside houses, and this led to an emphasis on physical barriers to prevent mosquitoes coming into the houses, as well as killing those that managed to gain entry. Wire gauze had been used to protect against flying insects since 1830 after the process had been industrialized and costs lowered. As early as 1830, there are anecdotal reports that a doctor from Connecticut had proposed that protecting houses in this way would protect against malaria (no reference found except a note in *Encyclopaedia Americana* 1835).

Records of the US patents show the popular use of wire gauze to protect people (US patent 281 502, 1883) and houses (US patent 415,913, 1889) against mosquitoes. Wire gauze was used routinely in the Mediterranean area to avoid mosquito biting at the end of the nineteenth century. The proposal that yellow fever transmission was through the *Stegomyia* mosquito in 1881 led to the use of physical protection against mosquitoes, as recommended by Carlos Finlay [9, 10]. There is, however, no evidence that hospitals or houses were equipped as he recommended. Physical protection was introduced in 1899–1900 at the Hospital Las Animas of la Habana after the role of the vector is proven, and its use was systematized by Reed and Gorgas [11–13]. The Sao Sebastian Hospital in Rio, specialized in treatment of yellow fever, was similarly equipped at the same time. The use of wire gauze spread very rapidly in all countries were yellow fever was endemic (known as Marchoux chambers in French Antilles). Finally, wire gauze was added to the British pharmacopoeia in August 1900.

Thus, by the time Celli ran the first large-scale trial against malaria by proofing houses with wire gauze, using gauze to protect against exposure to insect-borne diseases was well established. Celli’s trials [14, 15] have previously been studied, exploring the quantitative and statistical aspects [16], and for later developments in the use of wire gauze, including Manson’ personal trial [17, 18]. In summary, during the summer of 1899, Celli used wire gauze over the doors and windows of the houses of some railway workers working in a highly infected part of Latium. Veiled hats and gloves were provided to night-shift workers. Other railway workers were left unequipped. In autumn 1899, results were impressive: almost no inhabitant of equipped houses had contracted malaria, and those who did had not worn the proper equipment at night. Another experiment ran a year after was as successful, but more difficult to analyse, since quinine prophylaxis was used and chimneys also were protected (*Anopheles* of Latium has a trend to move up when in contact with an obstacle such as a wall). During summer 1900 too, Manson’s co-workers and Italian doctors Bastianelli and Bignami, lived in a similarly protected hut in Latium. None of them contracted the disease, in contrast to people living in similar but unprotected houses.

The two sets of trials demonstrate that very simple equipment, combined with rigorous adherence to using it, could dramatically reduce the incidence of malaria. Mosquito-proofing houses was subsequently used in many places in the world, meeting with a similar dramatic reduction (estimated 80–95%) in the number of cases of malaria [17, 19]. For those interested in the mosquito proofing windows and doors, Battesti a physician at Bastia hospital, Corsica, reported practical approaches in 1906. He tested out these approaches in trials in Furiani near Bastia in 1902, pointing out the need to also protect chimneys as well as the space between the roof and the walls. He also detailed prices for the protection of doors and windows, 30 and 15 francs respectively, thus equivalent to 120 and 60 euros, values to be compared with a maximum salary of 5 francs a day for a skilled worker [20]. The price explains in part the reason why the method did not spread fast and was rather a practice for protection of administrative buildings, such as barracks or railway station offices [17]. Lindsay discusses the reasons why the method has not been more used despite its apparent effectiveness, suggesting this was due in part to the cost, and in part because governments and physicians had always preferred unique, cheap and quick “solutions”, instead of considering alternative, moderately costly and partially efficient approaches [17]. Indeed, recent work has shown that the robustness of these procedures, for example in Mozambique, a pilot project suggested mosquito-proofing of houses impacted on malaria transmission. But still the difficulty remains the cost and thus the ability to scale up the method [21].

Mosquito-proofing of houses was completed with killing insects that have entered houses and remained indoors. Among the differences between 1899 and 1900 trials is the burning of pesticides in huts, and the walls painted in white to easily spot and kill mosquitoes [14, 15]. Killing indoor mosquitoes was necessary: huts were so badly constructed that *Anopheles* could enter through holes, despite protection of the openings. In addition, in the Latium area where Celli and Manson’s experiments were carried out, *Anopheles* present in peasant huts (illustrated by a photograph in [17], showing Manson’s hut), could remain alive during the entire autumn and winter (the tip of the roof is hot) and would lay eggs in spring. In the meantime, the mosquitoes could take several blood meals and, therefore, repeatedly infect inhabitants (Coluzzi, personal communication).

Procedures used to kill indoor insects had also been known for a long time. Burning brimstone had been a common practice to cleanse barrels, caves and ships from all kinds of animals—from rats to flies—since medieval times (see [22] for a literary description of the cleaning of a hold), and also in agriculture. It was also widely used in Brazil for fumigation of the houses of yellow fever patients, as shown by the movie made by Instituto Oswaldo Cruz in 1908. However, sulphur oxide could not easily be used in the presence of humans except in some rare cases, as in Algeria [23]. Despite the serious effects on local environment, sulphur candles were first used as a disinfectant, and were sometimes used against mosquitoes, for example, in the Panama Canal Zone.

Pyrethrum powder was the most frequently used pesticide against *Anopheles* and other dipterans. In contrast to sulfur, its history in Western countries is recent. Chrysanthemum powder had first been imported around 1830 from Persia and Dalmatia, where it was used against lice. Pyrethrum and Chrysanthemum powder prepared by grinding the roots and the flowers of several plants of the *Asteraceae* family, became widely used to fight bed bugs after 1840, as well as against many other insects [24]. Because insect plagues could devastate crops, insect powders have been extensively studied, particularly by the entomologists of the US Department of Agriculture. According to early reports [25, 26], pyrethrum powder proved very efficient as an insect killer, either used inside or outside buildings, though its efficiency was strongly dependent on the origin and nature of the plants used to prepare the insecticide. Pyrethrum was burnt or sprayed as a powder, or after WWI, as a liquid following extraction using alcohol or kerosene (Fly-Tox).

Based on its known effectiveness against many insect species, pyrethrum powder was used indoors against yellow fever vector [11–13]. The work of Gorgas in Cuba suggests that pyrethrum powder was one of the key components in the defeat of yellow fever in Cuba. Pyrethrum was used against domiciliary malaria [8, 23, 27]. However, this analysis has not found reliable quantitative data published before WWII and concerning the efficiency of pyrethrum on the indoor transmission of malaria. In contrast to the then proposed efficiency of pyrethrum, a post-war quantitative study of the effects of pyrethrum intensively spread within houses, carried out in Western Africa in 1947, failed to show any significant reduction either in the number of Anophelines or in the transmission of malaria [28]. Anyhow, the insecticide has largely been used as a local adjuvant in the context of composite strategies against the disease. Actually, the nearly 200 years old natural (and later synthetic) pyrethroids remain among the most used and important agents against indoor biting *Anopheles*.

## Combining local measures: *piccola bonifica*

The Italians developed an approach to malaria control in marshland with a set of procedures known as ‘*bonifica*’. *Bonifica* was first established by Royal Decree in 1883, which institutionalized long-standing land reclamation from marshes. The word itself could be translated as ‘reclamation’, or ‘sanitation’. Actually, *piccola bonifica* was the first anti-malaria strategy, including anti-malarial larval source management, to be assessed scientifically. The procedures had been originally tested in 1899 on the island of Asinara, offshore Sassari in Sardinia. Asinara was populated only by prisoners, their guards, and the personnel of the hospital. The idea was to eradicate anopheline populations by a combination of well-known procedures and to assess the consequences of the trial on the occurrence of new cases of malaria. Before the intervention, Fermi and a Dr. Tonsini [29] did a detailed study of the environment of the island and collected the most important information on the malarial areas identified, including health condition of prisoners and guards. The association of different kinds of interventions on habitat and malarial areas was then planned:

- Larviciding by petrolizing stagnant water; this intervention was made twice a month between June 1899 and the end of November 1899;
- *Anopheles* destruction, using a combination of Pyrethrum and Chrysanthemum powders as insecticides (sometimes also using calcium chloride mixed with sulfuric acid), in the houses and in the dormitories where prisoners lived;
- Physical protection of prisoners’ dormitories, applying thick muslin at the windows, and disinfecting the air every morning with insecticides. Every day during the experiment, the dormitories were inspected and a discussion held with the prisoners and guards.

In 1898, the year before the intervention, there were 99 new cases, 40 of whom were among people living in Asinara. After the intervention, all the houses were free from *Anopheles*, and no new cases of malaria occurred (the nine cases registered at the hospital were of people who had come from other parts of Sardinia or in whom there had been relapses).

These promising results reflected the main characteristics of the island: its position, the rarity of stagnant water, the absence of rivers and the small population to be protected. Fermi conceptualized the so-called ‘small reclamation’ or ‘*piccola bonifica*’, as achievable using the method he called ‘*disanofelizzazione idroaerea*’. This method, according to its original formulation outlined in 1899, consisted of destroying *Anopheles* larvae using salinization and petrol, with fortnightly interventions practiced on a large scale, and by eliminating their habitat through re-arrangement of the waterways, burying stagnant water, and covering tanks and wells. ‘*Piccola Bonifica*’ also involved killing *Anopheles* trapped in mesh or against glass, using asphyxiating gases.

Fermi and Tonsini first examined the features of outbreaks of *Anopheles* infesting the area, and reported the places on a map of the island. Thus, by the beginning in 1899, Fermi had introduced planimetry in his experiments against *Anopheles*, and this was used regularly to monitor the effects of the interventions that followed. Fermi’s methods were then applied systematically in many malarial areas (such as Sassari, in Sardinia), together with those proposed by Grassi and Celli, respectively physical protection and prophylaxis using quinine.

Fermi and Tonsini’s paper received a great deal of attention, and translations into German, English and French were published and discussed. Similar trials followed in other countries. For example, in the Andaman Islands penal settlement, mosquito-killing, bed-nets and combustible pastilles, prophylactic administration of quinine, keeping the population in a good state of general health, and reducing time spent in highly infectious areas was associated with a marked reduction of malaria cases over several years, in malarial conditions more aggressive than those on Asinara Island [1, 2, 30]. In 1903, Ernest Edwin Waters gave a detailed account of the measures taken to reduce malaria and some of the formal experiments designed to assess their effects. Thus:

*37 women* [prisoners] *selected from all classes were placed under mosquito curtains, going under them at dusk and coming out in the morning. Their occupation, health, and food in no way differed from any other section of the jail. The remainder of the jail population was divided into two classes. To one class 20 grains of quinine were given in two successive days; to the other no prophylactic issue was made. The effect was most marked. In Class A, in which the women slept under mosquito nets, there were 1007 admissions per 1000; in Class B, who received quinine, the admissions were 2421 per 1000; and in Class C, who received no quinine, they were 4177 per 1000* [30].

With the knowledge that insect vectors carried the disease, a variety of strategies were tried, and all aimed at reducing the frequency of contact between humans and the vector, and aimed at reducing the number of mosquitoes by killing both the adult vector and the larvae. The methods were adapted to each insect and each local situation, but procedures were largely the same. The important point in the case of *piccola bonifica* is that it raises once more the role of social factors in combating malaria: all the proposed measures can be carried out by local communities, provided they have learned the methods and understood the benefits they could expect by applying them [3, 4, 31]. However, with the exception of isolated areas such as the island of Asinara, the long-term effectiveness of *piccola bonifica* was not, or rather could not be, estimated quantitatively. Fermi declared Sassari a mosquito-free city, but the Rockefeller Foundation (RF) established one of its stations in Sassari in 1924 because it was then heavily affected by malaria. Thus, when the anti-malaria protocols used in 1924–1925 were compared, the efficiency of *piccola bonifica* appeared significant only locally and through permanent struggle against the vector [3, 4]. However, it is worth noting that Swellengrebel, a prominent member of the Malaria Commission of the League of Nations, in an attempt to extract from the reports which reached the Commission a set of partially efficient measures which could be applied by a majority of people in a majority of places, proposed rules very similar to *piccola bonifica* [32].

## Large scale larvae control programmes

Various anti-larval procedures were tried after 1900. The success met by Gorgas and Reed with yellow fever was due in part to the destruction of *Aedes* larvae [13]. Some of these large-scale programmes, such as alternate irrigation of fields every 2 weeks to kill developing larvae, met with some local success in Algeria [33]. Putting petroleum on water was also used in many countries to kill *Anopheles* and other insects’ larvae; but it had to be repeated too frequently to be useful. In addition, local farmers complained that treated water had become unpalatable to cattle, and this limited the use of this approach. Between the two World Wars two main anti-larval procedures were developed, both largely under the auspices, or with the support, of the International Health Bureau (IHB) of the RF [34]. The first is the use of Paris Green (Copper acetoarsenite), which prefigured large-scale use of chemical insecticides; and the second is the use of larvivorous fish, one of the first examples of ‘biological warfare’ against malaria. In both cases, the anti-malaria strategy relied on an initial scientific analysis of the local malarial complex [35] (equivalent to a present ecosystem) followed by the nearly exclusive recourse to anti-larval procedures, thus minimizing the administration of quinine. In the opinion of IHB staff, both strategies were clearly aimed at controlling malaria using a single, or a dominant, anti-larval approach. These became obsolete after WWII, although larval management can still complement core anti-malarial strategies, at least under particular circumstances.

## Insecticides and larvicides

*Anopheles* larvae swallow particles floating on the surface of water, so if these are poisonous, they die; and if the poison is the right size and floats, then the intention is the effect would be specific to *Anopheles* larvae and the poison would not kill other animals or damage the environment.

Roubaud, an entomologist at the Institut Pasteur, appears to have been the first to follow that logic, and to use trioxymethylene (trimer of formaldehyde) as particles spread on the surface of water to kill *Anopheles* larvae. He reported an immediate and large reduction in the number of larvae within days following spraying [36]. That success prompted the US Health Service (USHS) to extend the investigation of insecticides. The poison had to be sprayed as particles which stay on the surface of water and small enough to be swallowed by larvae. A number of toxic substances were tested in the laboratory. In short, known doses of the poison mixed with road dust as a carrier were sprayed on the surface of water in Petri dishes; a known number of larvae was added and the kinetics of their death was monitored.

In 1921, Paris Green was identified by Barber, an expert at the US Health Service, as the most efficient poison: it could kill larvae at the smallest lethal dose applied, which was 2 particles/larva/4–5 s of feeding [37]. Based on the results of laboratory experiments, powder of Paris Green was sprayed onto ponds infested by anopheline larvae. Twenty-four hours after spraying, 98–100% of the larvae had been killed. Finally, Barber specified the amount of Paris Green to be sprayed: 10 cm^3^ of the larvicide mixed with 100 cm^3^ of road dust were sufficient to kill all larvae over a surface of 400 m^2^. Spraying had to be repeated every 10 or 15 days. The possibility of chronic poisoning of animals and humans was considered but dismissed [37]. Moreover, Paris Green was cheap—about 22 cents a pound in 1923. It could easily be sprayed either manually of even from a plane, depending on the area to be treated [38]. A very successful field test was carried out during the 1922 season in Lake city (Florida) [39, 40]. Several further trials were carried out in the USA: the reduction in the number of *Anopheles* larvae was in the 98–100 range. However, this approach remained unfamiliar to Europeans before 1925 [41].

What is Paris Green, that “wonder drug” as Lewis Hackett, the RF executive in Italy, named it in 1925? Paris Green was not a new comer in the world of pesticides. Arsenic and arsenic derivatives have been used in agriculture since the middle of the eighteenth century in attempts to kill insects [42] capable of ruining seeds and crops. Results were uncertain and in any case mediocre [43]. Despite poor results and toxicity, arsenic oxide continued to de routinely used in agriculture particularly to protect seeds from mildew. Arsenic derivatives have been used instead of arsenic oxide since the end of the eighteenth century, particularly complexes of copper (or lead) with arsenic [44]. The first such compound had been prepared by Scheele (1742–1786) in 1778, but was mostly used as a green stain for wall papers. Unstable and highly poisonous, Scheele green was replaced in 1816 by a better-defined and more stable substance, the emerald green aceto-arsenite of copper, known as Schweinfurt green [45]. The chemical was easily prepared by boiling together equal parts of copper sulphate and arsenic oxide in water, adding potash and washing the precipitate with acetic acid. Highly toxic, the stain was used to kill rats in Paris sewers, which explains it’s given name of Paris Green. In 1866, Paris Green was sprayed on plants (particularly cotton in the USA) to fight mildew in association with copper sulphate. In 1868, it became widely used to combat cotton and potato pests [46]. From that time on, Paris Green became a pesticide frequently used in agriculture and also as a toxic paint to protect wooden boats from algae and mollusks. That long history and its proven toxicity explains well why Paris Green was on the list of molecules tested by Barber and Haynes in 1921.

At the moment Hackett was sent to Italy to fight malaria, the International Health Bureau (IHB) of the RF was convinced from its experience with yellow fever that the only way to control both diseases was a systematic anti-larval programme [47]. Paris Green is not mentioned in the Rockefeller archives before 1923, but the International Health Bureau soon took advantage of the results obtained by the US Health Service. Actually, it was the use of Paris Green as a larvicide by the RF for the first time in Europe in 1924–1925 by Hackett in Sardinia, which gave the agent its reputation: Paris Green was the long-awaited ‘wonder drug’ [7, 48].

Hackett and his Italian associate Missiroli, had several test sites in Italy, but they mainly focused on Porto-Torres near Sassari, which was still a highly malarial area despite *piccola bonifica* and the ready availability of quinine through a local dispensary. The doses and the protocol defined by Barber were used [41]. Hackett actually obtained identical results to those of Barber and Haynes in the USA. Results are reported at length in Hackett’s report to the first international congress of malariology (1925) and in reports to the International Health Bureau. In short, Paris Green powder was well mixed with thin road dust (ratio 1:100) and sprayed using hand blowers. Whatever type of water is treated (pool, ditch, stream and river), 96–100% of larvae were killed overnight (from an average of 300 larvae/square meter to zero) by a single spraying of Paris Green powder. The nature of banks and that of the vegetation in and around waters (clean, grassy, reeds, bush and cane) did not influence the results, In the example detailed by Hackett, after spraying a total length of 6 km of the river Turitano, which flows through Porto Torres, the number of anophelines declined markedly—an average 250-fold decrease. Similar results were obtained in Calabria (Bianconovo). The cost of making an area mosquito-free was estimated as 7–11 US cents per capita [48]. In addition to low cost, Hackett pointed out the following advantages of the approach: no highly trained employee is needed for that work; when properly used, neither acute nor chronic poisoning is noted; and treated water could be drunk by cattle. However, Hackett pointed out it is probably safer to use larvivorous fish (see below) to kill larvae in wells and water for human use, for example, in places where water is limited in summer (Calabria). Finally, Paris Green specifically killed *Anopheles* larvae and no others. By comparing their results to those obtained using other methods, such as *Piccola bonifica*, Hackett and Missiroli came to the conclusion that only Paris Green was rapidly and profoundly effective [49]. Based on preliminary results obtained in 1924 by Hackett and Missiroli, the Italian government introduced in 1925 instructions for the use of Paris Green [50].

Several countries adopted Paris Green after these results, including the Philippines, Palestine, the Netherlands, Brazil and Puerto-Rico. In France, results obtained in Corsica in 1925–1931 confirmed Hackett’s results [51, 52]. Officials of the International Health Bureau exulted: “*Paris Green was judged to have ‘proved a highly effective weapon…. The malaria incidence at Fajardo (Puerto*-*Rico) has been steadily reduced and no longer plays a major role in producing disability in the community*” (RF annual report 1927 quoted by Stapleton [49]). Thus, prophylactic administration of quinine could be reduced to a minimum in treated areas, a major change in anti-malaria strategy and a move towards the use of a single approach to control malaria. Toxicity was never considered a problem.

Although use of Paris Green spread rapidly, limitations soon appeared. In particular, the need to spread poisonous dust every 2 weeks during the period of larval development was a clear limitation, particularly where large areas have to be treated (extended marshes, for example). As a consequence, and to the disappointment of the RF, use of Paris Green could not always be scaled up to large areas, although aerial spreading was used to some extent [53]. Special boats also were built to reach and treat remote infected areas in marshes [52].

Paris Green was widely preferred as a larvicide by experts in the International Health Bureau of the RF, until it was replaced by insecticides particularly by DDT after 1944. Its use was a genuine success. Brazil had eliminated *Anopheles gambiae* by 1940 in large areas of the country following the introduction of Paris Green in the late 1920s [47, 54, 55]. Similarly, Egypt eliminated *An. gambiae* in Upper Egypt and Cairo using the same strategy in the early 1940s [56]. The RF’s policy and Hackett’s contribution had thus met with an enormous success. Paris Green remained the flagship of the malaria control policy of the RF’s International Health Bureau. Moreover, it reinforced the links of the Foundation with chemical industries and paved the way for the extensive usage of DDT after 1943, particularly in areas in which the Foundation had had longstanding involvement [47, 57].

## Larvivorous fish

*Gambusia* are the best known among larvivorous fishes used as anti-malarial interventions. These fishes are naturally occurring in Northern Mexico, Southwestern USA including North Carolina and Virginia whence they appear to have originated. *Gambusia* is a large genus belonging to the Poeciliidae family and of about 40 species. Of those, *Gambusia holbrooki* and *Gambusia affinis*, respectively Eastern and Western *Gambusi*a, were declared anti-malarial agents at the turn of the twentieth century ([58], decision by the US Department of Agriculture).

*Gambusia* first attracted the interest of zoologists because it is a viviparous fish, long before the discovery that *Anopheles* are vectors of malaria. After 1900, the idea that *Gambusia* could be used against mosquitos slowly emerged, meeting with a retrospectively reconstructed memory that fishes had been used for a long time in Barbados to clean water off “little worms at the surface”, presumably larvae. Seale in 1905 probably was the first to advocate the use of *Gambusia* because the local practice was associated with a low prevalence of malaria in Barbados [59]. The number of publications reporting the use of *Gambusia* as anti-malarial in many parts of the world grew steadily and “exploded” after 1920, largely because of the introduction of Gambusia in the programmes of the International Health Bureau. As with Paris Green, the use of the fish was well known in the USA and its sphere of influence, but relatively ignored in Europe, until after WWI and the introduction of Gambusia in Spain (1921), then in Italy (1923) and Corsica (1924), in the context of the International Health Bureau’s European actions.

*Gambusia* are notoriously voracious fish, eating any kind of small prey, including other *Gambusia*. *Anopheles* larvae are a very minor part of their diet. Records show that the use *Gambusia* against malaria was proposed in 1903–1904 in the context of their acclimatization to Hawaii’s climatic conditions [59]. An anti-malaria project itself was discussed in 1908, a project extended to Philippines in 1913. No quantification of *Gambusia* effect on larvae or *Anopheles* population was reported at this stage [60]. However, it appears that some trials had been carried out since as short descriptions published in medical journals after 1920 were located, when *Gambusia* were introduced in Europe. These publications suggest that killing larvae was efficient, but results were not provided in these reports. This anyway led to the registration of *Gambusia* as anti-malarial agents in 1919 [58].

Few field trials containing quantitative data have been reported in details. There were two: one was a 6-year long experiment carried out in Corsica between 1925 and 1931, and another conducted by de Buen in Spain at the same time. The first trial appears to have been a ‘laboratory experiment’. It was carried out by Emile Brumpt, a distinguished parasitologist at the Faculty of Medicine in Paris, who was in charge of the field trial under the auspices of the RF. First, the proper species of *Gambusia* was selected. This involved taking account of the biology of the fish, and the angle of the head to the surface of the water where the larvae live, so that salinity and calm water renewed without pumping were compatible with *Gambusia* breeding. Selected places were seeded with *Gambusia holbrooki* a year before the trial. Fishes proliferated.

Brumpt designed an experimental protocol to assess the effectiveness of *Gambusia holbrooki* against larvae [61]. In a channel close to Bastia, a place known for high endemicity of malaria, he placed three small fishing boats. Boat (A) was partly sunk in the channel, with water inside freely accessible to all kinds of fish; a second boat (B) remained afloat but the water inside was made accessible only to small fish like *Gambusia*; a third boat (C) was left floating, contained water, but water inside was not accessible to fish. 300–500 larvae per square meter were recovered from the channel and from boat C. No larvae were recovered from A and B. Brumpt concluded:

“*Il est difficile d’imaginer une démonstration plus frappante du rôle prophylactique de ces poissons larvivores”.* [It is difficult to imagine a more striking demonstration of the prophylactic role of these larvivorous fish].

Reports to the RF and to the French administration written by Brumpt’s collaborators in Corsica, Coulon and Sautet who ensured the follow up of the experiment, were less categorical about the effects over the longer term. Summarizing 5 years’ use of *Gambusia* in 16 different locations (canals, estuaries and rivers) over Corsica, they concluded that a 300- to 500-fold decrease in the number of larvae, thus a nearly complete disappearance, correlated with the presence of *Gambusia*, particularly when the latter was perennial. A weaker decline (10- to 50-fold) in the number of adult insects was also recorded in the three well studied places [62].

The second trial was carried out under field conditions in a highly infected area of Spain, close to the Portuguese border. De Buen ran experiments in five distinct parts of the province of Caceres [63]. He had obtained *Gambusia* from Dr. Sella in Madrid, who received them from the American Red cross in 1921. A hatchery was established in Talayuela (close to Caceres). Conditions for optimal breeding in the local conditions are precised. *Gambusia* were seeded in tests areas by lots of several hundreds. Seeding had to be repeated every year. All types of waters supporting the development of larvae have been tested (ponds, rivers, fountains, wells, lakes, puddles). De Buen observed a decline of 30–96% in the number of larvae, variation in numbers remaining unexplained.

De Buen’s trials are particularly interesting since he compared the anti-larval effects of Paris Green with those of *Gambusia* and of a combination of the two. He also tried other anti-larval agents (Stoxal—which is trimethoxyethylene mixed with dust, studied by Barber (some tests of the larvicide “Stoxal”, Public Health reports, 42, 1997–2004), and Leron, a pesticide which proved toxic to fishes and was rejected), but the results obtained on larvae with these agents were not presented. In De Buen’s hands, Paris Green yielded the same results as in Italy or Corsica, provided the insecticide was sprayed every 7–10 days. A combination of the two methods (Gambusia first, followed by insecticide), an experiment aimed at decreasing the frequency of spraying, did not improve the results. The effect on adult *Anopheles* populations is not mentioned. De Buen confirmed that Paris Green was specific for *Anopheles* larvae and not toxic to the rest of the fauna.

Several limitations to the use of larvivorous fish were noted. *Gambusia* do not always breed well. Except in permanent rivers and marshes, one needs to ‘seed’ waterways several times a year, particularly before larvae develop. That implies the need for technical support for fish production, hatchery and transportation of fragile animals, all conditions described in their papers. Coulon, Sautet as well as Buen concluded that *Gambusia* were indeed valuable within the context of a global anti-larval strategy, but that they should always be used in combination with other methods. Medical and colonial literature shows that Gambusia have been introduced and tested in most of parts of the British and French colonial empires, with the same, locally dramatic, but most often temporary, successful decrease of larvae numbers.

These experiments provided some evidence, as early as 1930, that *Gambusia* may be a helpful approach to control malaria, but there were clear issues with feasibility and it was thus unlikely to be a decisive approach under field conditions. Despite this, the World Health Organization (WHO) has promoted the use of larvivorous fish as an environmentally friendly alternative to insecticide-based interventions for malaria control. A WHO-sponsored interregional conference on malaria control in 1974 reported that “the utilization of larvivorous fish, mainly *Gambusia* or suitable local species, is the only practical measure that can be recommended where applicable, as in lakes, ponds, pools, wells, rice fields” [64]. A 2001 regional meeting in Kazakhstan recommended that more studies on larger numbers of local larvivorous and phytophagous fish be undertaken in different eco-epidemiological settings in that region, and that the search for effective larvivorous fish should continue [65**]**, and even the Global Fund funded country larvivorous fish programs until 2006. However, a recent Cochrane report recommended that “*before much is invested in this intervention, better research is needed to determine the effects of introducing larvivorous fish on adult Anopheles populations and on the number of people infected with malaria. Researchers need to use robust controls with an adequate number of sites*” [66]. Moreover, Gambusia is far from being environmentally friendly. It is worth noting that the voracity of *Gambusia* is such that the fish could destroy large parts of the fauna of rivers and lakes where they have been introduced, to the point of being considered as pest fishes [67].

## Impact of anti-larval interventions on malaria endemicity

The data from Brumpt, Hackett, Missiroli and De Buen, referring to different locations in Italy, Corsica and Spain, indicate that Paris Green and *Gambusia* were truly effective against larvae. Consequently, a marked reduction in the number of adult *Anopheles* populations was noted in a radius of several hundreds of meters distant from the treated area, although re-infestation from poorly accessible areas was recurrent. The anti-larval strategy of the RF appears thus a genuine success; this was indirectly supported by evidence that the density of *Anopheles* rapidly increased as soon as *Gambusia* disappeared or spreading of Paris Green was discontinued.

The consequence of the reduction of anopheline numbers on malaria was more difficult to assess. It appears that Brumpt in Corsica and Hackett who had carried out the first well designed trials in Italy, had moved a bit too hastily to conclude that it was effective in reducing malaria. It appeared that some decline had indeed been noted locally but this could not easily be quantified. In the absence of a valid population survey carried out prior to the initiation of anti-larval procedures, physicians could only note an unspecified decrease in the number of new malaria cases in some places. Actually, in his report to the New-York administration of the RF, Hackett claims that he noted an immediate decline in new malaria cases in Italy, but admits “*it was difficult to show consistent reduction in malaria infection rates in every district*” (report to RF 1928 quoted in [68]).

In Corsica, records of the meeting of the Corsica General Council of Sept. 25, 1929 (archives de l’Institut Pasteur, Fonds Brumpt), mentions that not a single new case of malaria was diagnosed in Porto-Vecchio (in the south east of Corsica). The city of Porto Vecchio and its surroundings were chosen as the site of a major trial because of the high prevalence (unquantified) of malaria. Ponds, marshes and rivers were treated with either Paris Green, or oil, or seeded with *Gambusia* after a careful analysis of each place, taking account of the average flying distance of *Anopheles*. The local eradication of malaria was achieved in a single year by two men at low cost. The report concluded:

«*Il n’est pas sans intérêt de dire que les premiers efforts de la mission Rockefeller avaient été suivis avec scepticisme par les populations qui n’escomptaient que de médiocres résultats de l’emploi des poudres insecticides et de l’action des gambusias; mais par suite d’un revirement qui est la meilleure consécration du succès obtenu, les habitants des divers hameaux voyaient avec déplaisir leurs gambusia destinés à peupler d’autres gites à anophélines. Ils craignaient en effet le retour des moustiques*». [It is worth noting that the communities were somewhat skeptical of whether the Rockefeller efforts would work, particularly the use of insecticides and of the action of gambusia; but they were all pleasantly surprised, and their opinion changed. Indeed, the inhabitants of the various hamlets were upset when their gambusia fish were taken away to populate other anophelines breeding sites. They indeed feared the return of mosquitoes].

It emerges from the report that local eradication of malaria was not due to Paris Green or to *Gambusia* alone, but was achieved through a combination of methods, the detailed map of which is kept in the archives of the Corse du Sud department in Bastia [52]. It also showed that success depended on permanent ‘seeding’ with *Gambusia*, repeated spreading of Paris Green and the use of oil on ponds. It could thus not be said that each of them was sufficient on its own to eradicate malaria.

However, evaluation of the decline in malaria cases remained rather subjective, “not a single case” being meaningless in view of the migration of populations which characterizes Eastern Corsica. Indeed, the physicians in charge of field control of malaria in Corsica [62] were less emphatic and concluded as follows:

*«Le paludisme a*-*t*-*il régressé dans ces régions qui ont bénéficié si largement de l’action anti*-*larvaire des* Gambusia*? Il est malheureusement impossible de façon précise car les éléments de comparaison nous manquent* (because precise earlier comparison data are missing). *Cependant, il semble bien que, pendant ces dernières années, il y ait eu une amélioration sensible de l’endémie paludéenne dans la plaine de Biguglia* etc.» [Did malaria decline in those areas which had so largely benefited of the anti-larval action of Gambusia? It is unfortunately impossible to conclude, because precise earlier comparison data are missing (…). However, it seems that, over the last years, a significant improvement of malaria endemicity has been noted in the plain of Biguglia etc.]. Gambusia persisted at Biguglia, near Bastia, until at least 1952 [52].

Results obtained on *Anopheles* populations, and subsequently (as Hackett and Brumpt claimed) on malaria endemicity, were criticized by epidemiologists at the RF, particularly by Putnam [69]. Part of her study dealt with malaria in Porto-Vecchio, claimed to be malaria-free in 1928–1929. Putnam identifies several serious flaws which invalidated the assumed causal association between the decline in malaria and the anti-larval procedures. First, she noted inadequate sampling of the population, since it included only people attending the dispensary (and the retention only of ill people) and children attending school. No numbers were reported, and only trends of the evolution of the malaria index can be deduced from the reports. As for the annual incidence of malaria, the physicians counted cases but did not pay attention to differences in the duration of observations: after correction, actually using the evolution of malaria obtained in non-treated areas the trend towards a decline in cases of malaria persisted; but the nearly complete disappearance observed in 1928–1929 may not have been caused only by anti-larval procedures, since decreases had been observed elsewhere in the absence of prophylaxis. Moreover, the physicians did not take into account the intense treatment of many patients with quinine, and an enhanced migration of the population to safer mountains in the summers of 1928 and 1929.

Putnam criticized Hackett’s work in Sardinia in identical terms, adding that decline was not apparent in every place tested; there were no controls; and it was well known that malaria morbidity fluctuated rapidly: The conclusion had thus been drawn too quickly: whilst the decline in endemicity may have been real, the success claimed by the two main research studies was far from established as due solely to *Gambusia* and/or Paris Green. Putnam supported continuation of the trial, and proposed the collection of additional variables to support more informative statistical surveys of the health of the populations. Unfortunately, Hackett had to slow down his field work in Italy after 1928 for political reasons [31, 68, 70], but kept working in Albania. In Corsica, field works were stopped around 1933 [52] due to a conflict between engineers and physicians following the departure of the RF in 1931. A detailed account of the statistical analyses reported by Putnam was published after WWII by Putnam and Hackett concerning Italy [71] and Albania [72]. After these initial observations and discussion, statisticians were routinely associated with the RF’s anti-malaria field teams [68–73].

Trials were continued in many places in Europe (Bulgaria, for example, supervised by Swellengrebel), Asia (particularly in India), America (Brazil) and Africa (particularly in Nigeria and Egypt). All these showed locally dramatic but temporary success. Occasionally, as in Brazil, the success obtained by Soper [54] persisted much longer. The RF kept records of a series of well conducted experiments with a view to progressively refining protocols that could be extended to a global strategy for malaria control. Paris Green had remained the wonder drug of 1925 and was clearly the preferred approach of the RF. Based on an extensive study of the RF archives, Stapleton [68] concluded that the easy shift to DDT in the 1940s was facilitated because of the depth of the “preparatory work” on Paris Green throughout the world.

## Modifying the environment: large scale engineering

Drainage and land reclamation from marshes use engineering strategies developed over thousands of years. Archeology and medieval studies have shown that they were primarily aimed at providing additional land for agriculture and cattle breeding, as well as for fishing. One of the consequences of these extensive procedures is that they prevented stagnation of water; and it had long been observed that fevers were less intense in well drained areas from Roman times to the eighteenth century [74].

At the beginning of the nineteenth century, water and marsh management were thus aimed at both preventing fevers and improving agriculture The discovery of the association of malaria with *Anopheles* brought a dual rationale for preventing malaria through changes in water environment: first, through increasing agricultural land, which improved the quality of life and the health of the population, and thus resistance to disease; and second, through a reduction in the number of *Anopheles* breeding places so decreasing the probability of malaria transmission. Water management strategies including modifying the habitat structurally, or others that manipulated the environment by draining streams for example, are thus generally assumed to have contributed to the gradual decline of malaria in Europe from the middle of the nineteenth century [75–77]. They almost certainly did, although perhaps in a more limited manner than often claimed. Changes in water flows and creation of new channels and water reservoirs also sometimes had detrimental effects and contributed to malaria epidemics, as through hydro-electric schemes in the USA, in the Panama Canal zone and irrigation in India to mention three well studied examples [6, 78, 79].

The ambiguity of the effects of water management on malaria endemicity, has amply been discussed in 1925, in the case of the Italian national policy of water management. Italians certainly were leaders in ‘sanitizing’ areas infected by malaria. An approach to the management of marshes named ‘*bonifica*’ was not originally aimed at fighting malaria [80] although a decree signed on behalf of the Bourbon monarchy in Napoli on 19 December 1817, quoted by Celli [81], associates *agraria bonifica* with prevention of malaria. Under the direction of the Ministry of Public Works and of local consortia, substantial funds were invested in land reclamation, extensive drainage, and construction of hydrological systems. According to Lutrario in 1928 [82], more than 500 million lire had been allocated for ‘bonification’ between 1883 and 1923, thus about 0.5% of the Italian budget. The law of 1923 developed the 1883 text into a complete theory called *grande bonifica.* In addition to large hydrological works (a network of interconnected canals and hydro-electric pumps), *grande integral bonifica* came to include screening of populations, larval control, administration of quinine, model housing, irrigation and major drainage, along with control of population movement, all points interesting to compare with the principles of present bonification explicited in the declaration of Abuja [83]. An anti-malaria component was thus added to existing schemes. A powerful administration coordinated all procedures, considered as a model for Mediterranean countries [3, 31, 68, 80, 82]. Mussolini himself, who had a genuine interest towards malaria, pointed to *grande bonifica* as the key issue for malaria control in Italy [31, 84]. Bonification was assumed to be responsible for a decrease in crude mortality rates from 500 to 63 per million per year between 1900 and 1923 in the areas in which it had been applied (Pô Valley, Agro Romano and the Pontine Marshes), while mortality rates elsewhere in Italy remained at about 400 per million per year, according to Lutrario [82] quoting the Directorate of Public Health.

In fact, the situation was far from being as clear as Lutrario proposed. The place of *grande bonifica* among other anti-malaria procedures has been discussed elsewhere [4, 31, 70]). There had been a steady decline in the prevalence of malaria over the previous 50 years due to a multiplicity of factors, among which it was difficult to identify the specific contribution of *bonifica*. No controlled experiments had been carried out. Finally, Swellengrebel ([3], p 168) stated that there was no convincing evidence that *Bonifica*, even in the extensive form of *grande bonifica*, was a genuine anti-malaria procedure. It had no direct effect on malaria endemicity, although it could have had an indirect one through the improvement of health and sanitary conditions, as Swellengrebel acknowledged. Since *bonifica* was a process that was complex, costly and endless (points already discussed by much earlier authors, such as Monfalcon in 1826 [74]), primarily aimed at economic development, it could not be recommended as *the one and only* model for malaria control. However, it may have contributed to the control of malaria under particular circumstances:

“*The exception of course are hydraulic works of such a kind as to prevent all larval growth, in which case its hygienic value may stand quite apart from its economic merit*” (Swellengrebel discussed in [3, 70]).

Swellengrebel did not deny the contribution of drainage and large engineering works to the decline of malaria in Europe (it was obviously part of Dutch policy in Holland, for example) and elsewhere worldwide. He merely stated that *grande bonifica* is not an anti-malaria strategy, as the Italian government had claimed, but promoted regional planning and development and only secondarily promoted the control of malaria [32]. In that respect, it is interesting to note that training centers for the population, such as the large one established in Nettuno (Latium) were equipped for teaching the practices of *piccola bonifica*, although the city itself is located in an area where *grande bonifica* had been established and was a showcase for its efficiency to show to malariologists from outside Italy. The two types of *bonifica* clearly were intermixed in that area, along with a significant improvement of housing, as shown by the photographs taken by members of the congress on malaria (Fund Brumpt archives of Institut Pasteur). As discussed by Snowden [31], it has from the beginning of the century been understood by Italian malaria workers, that education and the social and political environment were key factors in the success of anti-malaria campaigns. Thus, although official emphasis was placed on large engineering works for political reasons (political weight of the Ministry of Public Works, faced to a weaker Direction of Public Health, a situation which slowed down the work of the RF in Italy) rather than for their anti-malarial efficiency, a multiplicity of approaches has been used together, mixed to various extent depending on diverse local features. Nowadays, the necessity to associate local measures to large hydraulic works to avoid detrimental effects of the latter, has amply been documented in a 2005 WHO report [85].

Interestingly enough, whereas the League of Nations had promoted the development of diverse local measures in 1925 (similar to *piccola bonifica*), it suddenly switched in 1927 for no obvious reasons, to the promotion of *grande bonifica* as the best anti-malaria general policy [4, 75, 76, 80].

## Medical entomology and “species sanitation”

The examples of field trials documented above have mostly been carried out in Europe, particularly in the Mediterranean area. All of those carried out under the supervision of the RF have been first tested in the USA and in Latin America. A special mention should be made of some anti-malaria campaigns under tropical conditions. Partly and temporarily successful strategies used a combination of classical measures (quinine administration, cleaning, draining and derivation of water, grass cutting, oiling, physical protection, killing of adult mosquitoes) during the construction of the Panama Canal. A massive recourse to quinine was thought to be the solution for the Madeira-Marmore railroad in Brazil. In both cases, the anti-malaria procedures were massive, local and temporary, dictated as they were by the economic need to control malaria in the construction area during the periods required for completion of these works.

An interesting and novel figure emerged from these early works on malaria: that of the medical entomologist, and rapidly gained in importance. The medical entomologists were sometimes physicians, such as Lutz in Brazil or Darling in Panama; or, a decade later, a zoologist such as Swellengrebel in Dutch Indonesia and Europe. All physicians working on malaria had to become trained entomologists, at least in the specific field of dipteran vectors. At the turn of the century, the taxonomy of the vectors was well known [86–88], but numerous additional species and sub-species of *Anopheles* were described in many countries. Indeed, identification of species vectors was the main task of medical entomologists. Medical entomology developed rapidly as a specialized field of medical zoology and parasitology, not any more restricted to taxonomy [87, 88]. First largely centred on the role of water in the *Anopheles* reproduction cycle, medical entomology rapidly included various aspects of the biology of the insects grouped under the term ‘ethology’. More attention was paid to local “ecosystems” in relation to diseases (malaria, yellow fever, sleeping sickness, Chagas ‘disease). In addition, study of behaviour of each vector species (feeding habits and animal preferences, places for laying eggs etc.), led to a novel type of entomology-based strategy [88–90].

Swellengrebel exemplified the figure of a “medical entomologist”. The contribution of Swellengrebel in Dutch Indonesia was among the first and best thought through “entomological” approaches to the control of malaria. He actually led a zoological (or rather ethological) approach to malaria which he named “species sanitation” in 1919. The idea stemmed from the finding by Watson [91] that the principal vector of malaria in Malaysia, *Anopheles umbrosus*, had a very typical and restricted breeding area, specifically the thick shade of trees. By clearing wooden areas, the insect would not survive and malaria incidence should decline. The experiment was made. Indeed, clearing the jungle reduced hospital admissions for malaria and the splenic index [92]. Unfortunately, the restriction of breeding places for *An. umbrosus* left room for another anopheline species, *Anopheles maculatus* which bred well in cleared, exposed to sun, areas. That last point was not known when a similar to Watson’s entomological approach to the vector and the disease, was initiated by the Civil Medical service in Dutch Indonesia under the auspices of the Dutch ministry of colonies. The project was particularly developed by Swellengrebel [93]. Swellengrebel developed Watson’s idea after he had met him in Deli in 1913 (Indonesia) and started studying species breeding behaviour, and egg laying habits, and preferred type of water (fresh or brackish), thus developing an ethological approach to the control of malaria in Java and Sumatra [94]. Swellengrebel went to the Dutch Indies for a second period of research in 1917, this time with his bride, a schoolteacher, whom he trained in malaria research and entomology. They worked together on the new concept described above, the study of malaria transmission in the field, so recording breeding places, infected mosquitoes in houses, species of *Anopheles* and spleen sizes in the local populations. Blood from people with enlarged spleens was checked for parasites and quinine was given (under supervision of a medical doctor). An additional problem was the scarce information about the species of mosquitoes in the huge Indonesian archipelago. They thus characterized the morphological features of adults and larvae, and devised diagnostic criteria for them. They also tested their respective vectorial capacities. Soon, they found out which mosquitoes were important vectors in a given location. Over a period of 2 years, they worked in about 30 locations, mainly in Java and Sumatra. Their multiparameter study provided insights into differences observed in the distribution of malaria epidemiological features in those two islands. In particular, a survey of the different anopheline populations in Sumatra and Java allowed the identification of three dominant groups of vectors: one with a ubiquitous distribution, one living on hills, and one in a very particular ecosystem. Species sanitation could only be applied for the last group, consisting mostly of *Anopheles sundaicus* [at the time known as *Anopheles ludlowi*] and distributed along the northern coast of Java, in fish ponds with inlets of seawater [95].

In 1920, Swellengrebel published an overview of malaria surveys in Indonesia and its future prospects [96]. An important point is that the identification of the effective vectors, their levels of infection by *Plasmodium* and that of the infected people, restricted the surface to be treated by conventional practices. Thus, only some well-defined areas had to be treated to significantly reduce malaria incidence. This controlled approach, in which entomologists, engineers and physicians had to work together was applied to the northern coast of Java in 1919 and further developed thereafter (regular lowering of the water level to reduce breeding of *A. ludlowi*). A combination of classical measures as for *piccola bonifica* (draining, killing of larvae and adults, quinine distribution) was adapted to local conditions.

The consequences of these anti-malaria procedures were estimated through evolution of the spleen index. From 1925 to 1932, a significant decline of the spleen index (two to tenfold decrease, depending on the place) was noted [97, 98]. Cautiously, Swellengrebel concluded that at least in the case of an epidemic level, species-specific control along with prophylactic distribution of quinine to the population, reduced the prevalence of malaria tenfold, thus to an endemic level. Swellengrebel used the endemic level in his homeland in The Netherlands as the basis for controlled experiments in the field and an example for European malaria groups. One lesson he brought back home from Indonesia was the importance of brackish water, the favorite environment for egg laying by the local vector *Anopheles maculipennis atroparvus*. The limitations of species-specific control soon appeared in Indonesia. First, an anopheline species so far harmless could acquire *Plasmodium* and become dangerous; also, due to the sanitation program itself, the vector can change its behaviour and escape from sanitation [99]. In other words, species-specific control modified the original equilibrium between insect populations. Despite these limitations, species-specific control was undoubtedly useful in the difficult conditions of Southeast Asia. It was considered the model to follow by the French physicians who were responsible for control of malaria in nearby French Indochina [100]. Also, the reference to Swellengrebel’s, and the insistence on the importance of taking account of local conditions and of the biology of the insects is obvious in the 1925 report of the Malaria Commission of the League of Nations [3] and illustrates the dominant influence of Swellengrebel in the field of malaria studies in Europe at that time [93], as was that of Watson in South-Eastern Asia [101].

The application of the species-specific control concept did not always meet with success. It soon became limited to a geographical survey of the places where anti-malaria campaigns were likely to be most useful. The scientific surveys carried out by the RF were of this type. In a different way, the study of the behaviour of several phenotypical traits of anophelines paved the way to in depth biological studies, such as that of variants able or unable to transmit malaria, introducing to the genetics of that trait [90]. Also, breeding habits were tentatively used to divert anophelines to bite a target other than humans, leading to the notion of zoophilic *vs* anthropophilic variants of *Anopheles*. Some Lamarckian-oriented entomologists attempted to train *Anopheles* and mosquitoes to bite cattle rather that humans [102].

Zooprophylaxis (using, for example, rabbits, chicken, or cattle to divert *Anopheles*) had been advocated by Grassi. Following the latter, Brumpt in 1922 considered that diversion was due to the favorable environment provided by animal barns [103]. The presence of domestic animals around farms is mentioned as a malaria parameter in reports to the International Health Bureau). It was tested between WWI and WWII, particularly (according to League of Nations reports) in Rumania and Bulgaria (Danube delta). No quantitative evidence, however, could then testify of the efficiency of zooprophylaxis in the control of the vector, at least during the period of time considered in the present paper. Zooprophylaxis was then only assumed to be one among anti-malaria tools. Recent survey of the literature suggests that zooprophylaxis may be useful at least in certain locations, provided the dominant vector has a strong avidity for livestock and provided the latter is kept away from human sleeping places [104].

## Palestine: a neglected proof of concept?

By 1925, malariologists worldwide were aware that there was no single effective strategy for combating malaria. Some control of malaria had been achieved through bonification in some places in Northern and Central Italy. Some local success had been obtained in other countries, but, most often, results were local and unstable. No control of malaria had been achieved over a large territory. Partial successes could have been in part due to the general decline of malaria in developed and developing countries, more or less independently of human medical and technical choices concerning the disease.

Control of malaria did, however, happen elsewhere. One of the most informative example is probably Kligler’s work in Palestine. In most places (upper Galilee and the Jezreel Valley) this had resulted in nearly complete eradication of *Anopheles* and malaria between 1922 and 1926 [105, 106], success that was highly praised in the Malaria Commission’s final report, which outlines the principles of anti-malarial procedures in Europe [107].

Malaria had been common in the plains of Palestine. However, endemicity was extremely variable from place to place, and was thus considered by scientists in the Hadassah Medical School (Jerusalem) as a kind of mosaic of local, distinct, malarious areas. Thus, a careful analysis was made of the biological and physical features of each of these, with particular attention paid to water flows and stagnation, and the species of *Anopheles* present. These studies resulted in the decision to use sets of different anti-larval procedures adapted to local conditions. These combined minor surface drainage and deep drainage of water, land reclamation from marshes, the use of quinine as a prophylactic and to treat malaria cases, and adequate housing and education of the population. Every known anti-larval procedure was also used, either alone, or in combination, depending on the features of the area considered. They included alternate water flows, similar to those used by the Sergent brothers in Algeria, cleaning of water channels, and spraying of oil and Paris Green.

In his preliminary report dated 1925 [105], Kligler concluded that new cases of malaria in Palestine declined tenfold between 1922 and 1925. Actually, numbers had reached zero in most places by 1926 [106]. Anti-malarial efforts were continued later, a decision which prevented the development of some highly pernicious epidemics, such as that which affected other Mediterranean locations in 1925 [108]. Kligler’s book makes clear that his work with colleagues was largely inspired by work in the Panama Canal Zone, and by the ‘ecological’ and scientific strategy of the Rockefeller Foundation (Kligler himself had been a member of the RF). However, without disparaging the important work of Kligler, tertian malaria and tropical malaria peaked again during the winters of 1926 and 1927, according to data collected in Tiberias area [108, 109], a finding pointing to the need for permanent efforts to control malaria and avoid re-infestation.

Why was this approach more effective in Palestine than in Europe and elsewhere? The answer probably lies in the fact that anti-larval strategy had been methodically applied continually in Palestine in association with other procedures, whereas it had been discontinued in Italy and Corsica. Also, due to the geography of the treated areas, the risk of re-infestation from other areas was limited. Importantly, the anti-malarial campaign in Palestine had been incorporated within the overall Zionist strategy for colonizing Palestine, which meant that it was well funded and organized efficiently by the Jewish National Fund. British mandate authorities approved the project but did not significantly contribute to its realization [106, 110]. The success of the approach used in Palestine can be considered as a proof-of-concept in support of a science-based strategy, combining several distinct control measures, provided it is solid political will behind it, and adequate finance. It also shows that the simultaneous application of a number of partially efficient anti-malarial protocols could lead to control of the disease.

## Conclusions

Empirical strategies for malaria control were developed before the biology of *Anopheles* and the malaria cycle was understood. The prevention and treatment approaches established before 1890 proved effective, although adopting them at scale to large populations was problematic. Indeed, scientific knowledge did not contribute much to the development of these early anti-malaria strategies, but merely provided a rationale. The transformation of bonification into a deliberate anti-malarial strategy was a clear-cut example of a change in the explicit goal without an accompanying change in the strategies used.

Less than 30 years after transmission of malaria was understood, there are now a variety of methods for prevention of malaria that have been tested. With the exception of the large-scale use of pesticides introduced much later, nearly all components of present anti-malaria strategies have been defined, tested and their limitations understood before 1930. Prophylaxis with quinine was shown efficient at the individual level and for some clearly defined and organized groups such as soldiers or prisoners, but could not be scaled up to the level of general populations. This is described in a separate publication [1, 2]. This analysis concludes:

- Correct mosquito-proofing of houses was shown to be very efficient but could not easily be scaled up mainly because of the cost of the equipment. Physical protection of persons also was efficient, if properly used.
- Combination of local measures including local water management sometimes worked, but depended on many factors: the approaches needed to be locally relevant, locally applied, with involvement of the communities living there.
- Large scale larvae management, once the flagship of the Rockefeller Foundation, was through two main approaches. Larvivorous fishes such as *Gambusia,* initially thought as creating a long-lasting protection, did not deliver, and their use was progressively restricted to particular ecosystemic situations. Copper arsenite (Paris-Green) met with greater success and contributed to many regional successes and to the marked decline of malaria endemicity in several localities. The approach was limited by the need for frequent spreading and the poor accessibility of most malarial areas. The toxicity of arsenic, though mentioned, was not reported as a serious problem.
- There remain questions around large scale drainage and related engineering, flagship of Italian administration in the struggle against malaria. Did this really decrease the endemicity of the disease in the sanitized areas? Efficiency of large engineering works was widely questioned; it appears mediocre in the absence of additional anti-malaria measures.
- Biological approach of malaria control, first introduced in South-Eastern Asia by Watson and Swellengrebel, largely relied on distinctive biological features of the vector. It met with some local and temporary successes, and were limited due to the consequences of the changes introduced in the ecosystems.
- Finally, the rational combination of all of the above-mentioned methods on a well-defined malarial environment, led to an efficient, long-lasting, control of malaria, in Palestine for example and some places in Italy.

One lesson that can be drawn from these results is that successful bio-environmental strategies of all kinds are essentially opportunistic. This means that the present use of these or similar strategies (*Gambusia*, modification of environment for example) probably needs to be carefully be adapted to the specific biology of the local vector and local ecosystems. In most cases bio-environmental strategies take advantage of environmental conditions which are exceptional in one way or another. For example, success is most likely where vector breeding sites are unusually restricted in distribution and relatively easy to locate. Such conditions are often found in urban areas. They are not common in rural areas particularly in the humid tropics. They are especially rare where the terrain is difficult, and where the local vector prefers breeding sites that are scattered and shifting, as in the forests and hilly areas of Southeast Asia. As a matter of consequence, bio-environmental strategies may be successful there in some restricted and isolated areas, such as the Andaman Islands described previously. In other areas, the application of residual insecticides to the walls of houses or to bednets will normally be a more reliable option for malaria control authorities.

Some other conclusions are reached from the study of historical sources. Trials conducted in the early years of the twentieth century, whatever concerning quinine use, mosquito-proofing of houses or changes in the local environment, have been carried out on enclosed populations (soldiers and prisoners) or on particular cadres (railway workers for example). Most of the difficulties reported in the scaling up of these anti-malaria methods relates to them being acceptable to the general population. People had to be convinced of the beneficial effects of a long-term discipline of the repetitive actions needed for prophylaxis to become efficient enough. Rejection and negligence have been the dominant obstacles to the scaling up of these methods, whereas training and education appeared to be the solution.

There is probably agreement that a strong government commitment to administer programmes combined with sustained investment is important. In that respect, early field trials such as those carried out by Grassi and Celli, were supported by universities and by branches of national institutions. All other trials were conducted and supported by State institutions. In that respect, Italian administration was considered as a model for the Malaria Committee of the League of Nations [3]. Dutch [93] and British [111, 112] colonial administrations possessed strong anti-malaria components. The French largely relied on overseas Instituts Pasteur and their tight links with military physicians [87]. Powerful private institutions such as the Jewish Fund in Palestine, and the RF throughout the world, played a primary role. Rockefeller Foundation never acted alone but always in association with local authorities and state institutions. In many respects, the RF can be compared with the present Gates initiative [113]. The strategy of the RF progressively elaborated out of campaigns against hookworm and yellow fever since 1913, proposed a short-term help (which can be renewed once) to conduct anti-malaria campaigns following RF-defined field strategy and train people so that they become able to continue the work by themselves [34, 52, 76]. This implies the contribution of the states particularly concerning the financing, after the Foundation has left. It is interesting to note that the larvicides protocols used by RF have been designed by the US Ministry of health, and that of agriculture and fisheries, in continuation of their earlier involvement in agricultural entomology.

Finally, perhaps the significant novelty associated with the new scientific knowledge on malaria, concerned the introduction of rationalized local interventions, as emphasized Grassi, Fermi, Swellengrebel and Watson at the very beginning of the twentieth century, and their coordinated and controlled use by Kligler. Each of the strategies described has proven partially successful, but their rationale combination was a major move in malaria prophylaxis. The Malaria Commission, particularly in its second final 1927 report [114], muddled as it was with a choice between ‘hydraulic’ (engineering and drainage) and ‘medical’ approaches to malaria and fascinated by the war machine set up in Italy, may not have perceived the epistemological change introduced by these actors: malaria should be approached as a series of local problems. The teams mentioned above had understood that no unique strategy would control malaria; quite the reverse, only combined use of a number of partially effective methods could lead to adequate local control of malaria. To be efficient, an anti-malarial strategy based on a combination of methods should essentially be opportunistic and take into account the local conditions, and be designed only after a detailed study of the place to be treated, the preparatory study should include the biology of the transmitting agent, with the screening of mosquito species and sub-species to define the actual vector, its location and that of its breeding sites; its behaviour and life cycle; and the features of the transmitted parasite. It also needed to include a study of local climate, fauna, flora, hydro-geography (local management of water) and geology, not excluding traditional anti-mosquito procedures. Swellengrebel proposals of 1925 for future research on malaria prevention are a good summary of such a multidisciplinary approach to malaria prophylaxis. Such a preparatory work requires the participation of entomologists and that of other scientific specialists. The reports to the RF, along with Kligler’s studies, are indeed remarkable examples of studies of pathogenic ecosystems, although the word ecosystem may sound anachronistic. This biological approach is not entirely specific to malaria; a related strategy was initiated at the turn of the twentieth century for control of yellow fever and sleeping sickness or Chagas disease, as soon as the vectors of the diseases had been identified [88]. In no case has the study of the biological and physical features of an ecosystem pathogenic to humans been pushed so far as it was in malaria. In that respect, the genuine novelty of the scientific approach to control of malaria before the 1930s was that the biology of the insect and ecosystems were seriously considered in anti-malaria campaigns. In that sense, it is unfortunate to note, from later League of Nations reports, that too little attention was paid to those insights.
